# Artificial intelligence-driven approaches in pituitary neuroendocrine tumors: integrating endocrine-metabolic profiling for enhanced diagnostics and therapeutics

**DOI:** 10.3389/fendo.2025.1618412

**Published:** 2025-10-16

**Authors:** Aiping Zheng, Dan Tang, Huijuan He, Xinyu Liang

**Affiliations:** ^1^ The Quzhou Affiliated Hospital of Wenzhou Medical University, Quzhou People’s Hospital, Quzhou, China; ^2^ Department of Medical Oncology, Cancer Center, West China Hospital, Sichuan University, Chengdu, Sichuan, China; ^3^ Department of Pathology, The Affiliated Traditional Chinese Medicine Hospital, Southwest Medical University, Luzhou, Sichuan, China

**Keywords:** artificial intelligence, deep learning, pituitary neuroendocrine tumors, diagnostics, therapeutics

## Abstract

Pituitary neuroendocrine tumors (PitNETs) pose diagnostic and therapeutic challenges due to their heterogeneity and complex endocrine-metabolic interactions. Artificial intelligence (AI) enhances PitNET management through improved classification, outcome prediction, and personalized treatment. However, current AI models face limitations, including small, single-center datasets and insufficient integration of multi-omics or autoimmune-associated biomarkers. Future advancements require multicenter standardized databases, explainable AI frameworks, and multimodal data fusion. By decoding endocrine-metabolic dysregulation and its link to tumor behavior, AI-driven precision medicine can optimize PitNET care. This review highlights AI’s potential in PitNETs while addressing key challenges and future directions for clinical translation.

## 1 Introduction

Pituitary neuroendocrine tumors (PitNETs) are the second most common intracranial tumors, comprising approximately 15% of all intracranial neoplasms and representing the most prevalent neuroendocrine tumors in adults, with an estimated annual incidence of 3.9 per 100,000 individuals (1). These tumors exhibit diverse biological behaviors, including variable growth patterns and invasive potential. Clinically, PitNETs may present with mass effects—such as headaches and visual field deficits—or with hormone hypersecretion, leading to conditions such as acromegaly, amenorrhea, and galactorrhea (2). While most PitNETs are benign adenomas treatable with surgical resection, medical therapy, or radiotherapy, a subset exhibits therapeutic resistance, recurrent growth, or, in rare instances, metastatic potential (3).

Advancements in artificial intelligence (AI) have significantly reshaped the diagnostic and therapeutic landscape of PitNETs, particularly in the areas of radiomics, pathomics, pharmacotherapy, and surgical interventions. AI, often considered the driving force behind modern medical innovation, has enabled the integration of machine learning algorithms, deep learning models, and neural networks into clinical workflows, thereby transforming conventional diagnostic and treatment paradigms (4). Currently, AI applications in PitNETs primarily focus on medical image analysis and clinical decision support, utilizing machine learning (including neural networks and deep learning) as well as rule-based expert systems. AI-driven methodologies offer notable advantages, such as enhanced measurement precision, superior detail detection, reduced interobserver variability, and improved predictive modeling for disease progression and therapeutic response (5).

**Figure 1:**
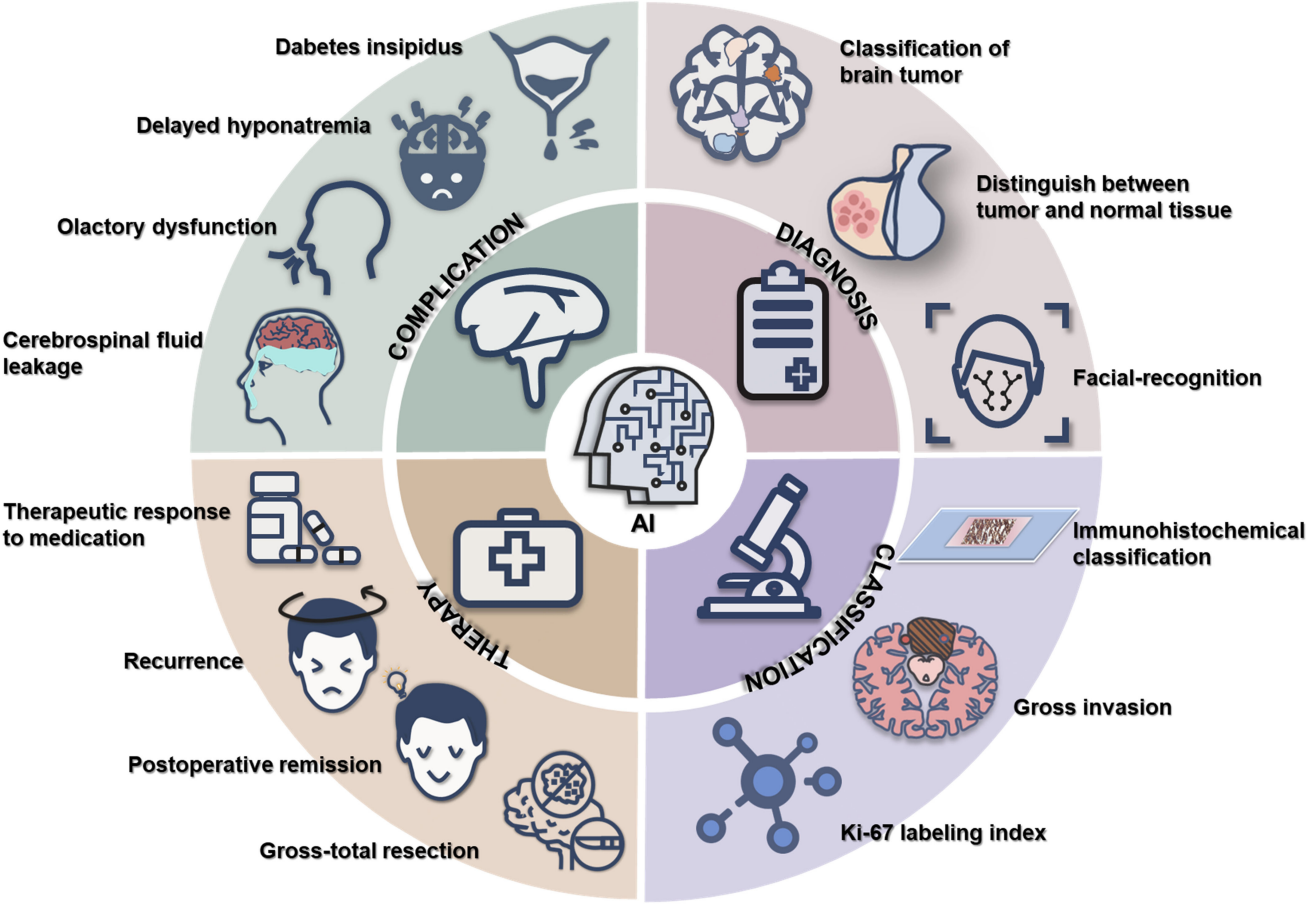
AI in the diagnosis, classification, therapy and complication of PitNETs.

Recent technological advancements have been extensively applied in oncological research, enhancing the accuracy of pathological diagnoses, prognosis predictions, and biomarker discoveries in malignancies such as breast, bladder, gastric, and lung cancers (6–9). In particular, AI has been increasingly integrated into the comprehensive management of PitNETs, especially in the optimization of surgical strategies. This review systematically examines AI applications in the diagnosis, therapeutic decision-making, and prognostic evaluation of PitNETs, while highlighting current progress and future directions in the field (**Figure 1**).

## 2 AI in PitNETs

Effective management of PitNETs requires timely diagnosis, precise classification and grading, personalized therapeutic strategies, optimized surgical planning, and long-term post-treatment surveillance to improve clinical outcomes. However, the heterogeneity in the biological behavior of PitNET subtypes poses significant challenges across the diagnostic and therapeutic continuum.

In recent years, AI has become a transformative tool in the diagnosis, treatment, and prognostic evaluation of PitNETs. AI-driven methodologies improve diagnostic accuracy and therapeutic precision, reduce healthcare costs, and facilitate seamless integration of information within clinical workflows. This chapter provides a comprehensive overview of the current applications and challenges of AI in the diagnosis, treatment, and prognosis of PitNETs, highlighting its potential to optimize patient management and support clinical decision-making.

### 2.1 Diagnosis and differential diagnosis

Accurate diagnosis of PitNETs requires a multidisciplinary approach involving pathology, endocrinology, neurosurgery, radiology, and oncology. However, the heterogeneous clinical presentations, radiological features, and histopathological characteristics of PitNETs present significant diagnostic challenges, often leading to inconsistencies across specialties. The integration of AI-assisted diagnostic tools has the potential to reduce cognitive biases and improve diagnostic precision.

AI-driven models have been widely applied to distinguish pituitary tumors from other intracranial neoplasms, such as meningiomas and gliomas, using deep learning algorithms trained on MRI datasets. Advanced classification models, including DenseNet, PDSCNN-RRELM, SIBOW-SVM, and MobileNetV2, have demonstrated high accuracy in brain tumor classification, thereby enhancing clinical decision-making, improving diagnostic precision, and optimizing patient outcomes (10–14).

Beyond tumor differentiation, AI has also been utilized to distinguish non-neoplastic pituitary lesions, such as pituitary inflammation, from non-functional PitNETs, thereby reducing the risk of unnecessary surgical interventions (15). Moreover, differentiating cystic PitNETs from Rathke cleft cysts remains a diagnostic challenge due to overlapping imaging features. A composite model combining MRI-based artificial neural networks (ANNs) with semantic analysis has demonstrated superior diagnostic performance, achieving an area under the curve (AUC) of 0.848 (16). Further advancements in AI-driven imaging analysis include the development of neural networks and deep learning algorithms for precise segmentation of both normal pituitary structures and tumor regions, thereby assisting clinicians in making more informed diagnostic decisions (17).

AI has been increasingly utilized for the early detection of acromegaly by integrating three-dimensional (3D) imaging and machine learning techniques to analyze facial morphology. Acromegaly is characterized by distinct craniofacial alterations, such as mandibular prognathism, jaw elongation, malocclusion, increased mandibular angle, nasal widening, and lip thickening or eversion. AI-based models facilitate the automated tracking of these facial changes, enabling earlier diagnosis and timely intervention, which can prevent irreversible complications associated with excess growth hormone secretion (18).

### 2.2 Tumor classification and grading

The current World Health Organization (WHO) classification of PitNETs is primarily based on the immunohistochemical expression of specific transcription factors—T-box pituitary transcription factor (Tpit), pituitary transcription factor 1 (Pit-1), steroidogenic factor 1 (SF-1), GATA binding protein 3 (GATA3), and estrogen receptor alpha (ERα)—in addition to adenohypophyseal hormones such as adrenocorticotropic hormone (ACTH), growth hormone (GH), prolactin (PRL), β-thyroid-stimulating hormone (β-TSH), β-follicle-stimulating hormone (β-FSH), β-luteinizing hormone (β-LH), and the glycoprotein hormone α-subunit (19).

Recent studies have employed machine learning (ML) techniques to improve the preoperative classification of PitNETs by analyzing radiomic features extracted from MRI scans. Various ML models, including support vector machines (SVM), k-nearest neighbors (KNN), and naïve Bayes (NB), have been developed to predict immunohistochemical subtypes. Among these models, SVM demonstrated superior performance, achieving an AUC of 0.9549 in distinguishing the Tpit, Pit-1, and SF-1 subtypes (20). Additionally, AI models have been utilized to predict hormone secretion profiles, categorizing PitNETs into non-functional adenomas, GH-secreting adenomas, prolactinomas, ACTH-secreting adenomas, plurihormonal adenomas, FSH/LH-secreting adenomas, and TSH-secreting adenomas. However, the Gaussian process (GP) model showed limited accuracy (AUC = 0.711), likely due to imbalanced sample distribution (21). Similarly, ANNs displayed suboptimal performance in distinguishing prolactinomas from other adenoma subtypes (AUC = 0.74) (22).

Most AI research has concentrated on binary classification tasks to enhance diagnostic workflows. Notable applications include SVM-based identification of non-functional PitNETs (23), Pyradiomics-assisted detection of silent corticotroph adenomas (24), and multi-sequence logistic regression (LR) models for distinguishing somatotroph from gonadotroph PitNETs (25).

Beyond classification, PitNET grading utilizes the Trouillas system, which integrates gross invasion and proliferative markers (mitotic count and Ki-67 labeling index) to categorize tumors into five prognostic grades (26). Assessment of gross invasion relies on advanced neuroimaging and neuroradiological expertise, with AI-driven models showing potential to enhance diagnostic accuracy and reduce observer bias. Several studies have applied convolutional neural networks (CNNs) and SVMs to evaluate cavernous sinus invasion using preoperative MRI, achieving AUC values of 0.98 and 0.871, respectively (27–31). Additionally, a deep learning model trained on high-resolution MRI images (1 mm slice thickness) demonstrated strong performance in preoperatively predicting cavernous sinus infiltration (AUC = 0.89), providing improved assessment of tumor depth and carotid artery involvement (32).

AI-based models have also been employed to assess tumor invasiveness from various anatomical perspectives. For instance, Feng et al. assembled 1,413 coronal/sagittal MRI scans from 695 pituitary adenoma (PA) patients, stratified into invasive (n=530) and non-invasive groups (n=883) based on surgical findings of sellar floor invasion A 100-image external test set was randomly selected, with the remaining 1,313 split 4:1 into training/validation sets. CNNs have been trained to detect PitNET infiltration of the sellar floor with high diagnostic accuracy (AUC = 0.98) (31). These advancements not only enable objective evaluation of the Trouillas score but also inform surgical decision-making, optimize follow-up strategies, and enhance long-term management by facilitating more personalized and cost-effective patient care.

PitNETs exhibiting a mitotic count exceeding 2 per 10 high-power fields (HPF) and a Ki-67 labeling index (LI) greater than 3% are indicative of more aggressive clinical behavior (26). However, the manual evaluation of these parameters by pathologists is inherently subjective and susceptible to interobserver variability. AI-assisted quantification offers a standardized and objective alternative, reducing assessment bias and enhancing diagnostic consistency. Recent studies have investigated MRI-based predictive models for estimating the Ki-67 LI in preoperative PitNETs. Lorenzo et al. analyzed a total of 89 patients who underwent an endoscopic endonasal procedure for PA removal with available ki-67 labeling index were included. From T2-weighted MR images, 1128 quantitative imaging features were extracted. Shu et al. collect MRI data from 234 of these PA patients to develop ML models to predict Ki67LI status, and ML models were tested on 27 PA patients in the clinical setting. ML algorithms trained on texture features derived from T2-weighted MRI demonstrated superior performance. KNN models achieved an optimal operating characteristic receiver operating curve (OC-ROC) of 0.87. CNNs also yielded promising results (33, 34). This allows clinicians to assist in determining the Ki-67 status through non-invasive tests. These AI-driven methods enable more accurate preoperative tumor classification, facilitate personalized surgical planning, and support the development of cost-effective, risk-adaptive follow-up and long-term management strategies.

### 2.3 Therapy and prognosis

#### 2.3.1 Surgical decision-making

According to the Endocrine Society Clinical Practice Guideline, transsphenoidal surgery (TSS) remains the first-line treatment for most PitNETs requiring intervention, except for prolactinomas, which are primarily managed pharmacologically (35). The endoscopic transsphenoidal approach demands considerable technical expertise, driving the development of AI-assisted surgical guidance systems to optimize intraoperative performance. These AI-enhanced protocols have shown promising results in improving surgical precision and efficiency, with preliminary evidence suggesting potential benefits for patient outcomes (36, 37).

Precise intraoperative differentiation between tumor and non-neoplastic tissue is critical for maximizing resection extent while minimizing recurrence risk. A recent study developed a deep learning model based on a Wide-ResNet architecture, trained on 4K ultra-high-definition intraoperative TSS images. Among the 605 static images and the cropped 117223 patches included in the training set, 58088 were labeled as tumors, while the remaining 59135 were labeled as non-tumorous tissues. The classifier achieved an accuracy of 76.8% in distinguishing PitNETs from adjacent structures. This advancement enhances surgical precision, particularly for less experienced neurosurgeons (38). Additionally, avoiding injury to critical structures—such as the internal carotid arteries and optic nerves—is paramount during TSS, as inadvertent damage may result in vision loss or life-threatening hemorrhage (39). However, due to their posterior positioning relative to the sphenoid bone, localization often relies on subtle osseous landmarks (40). To address this challenge, PitSurgRT, a multitask neural network, was trained on 635 frames obtained from 64 endoscopic pituitary surgery videos to provide real-time anatomical segmentation and surgical landmark detection. In clinical validation involving 15 neurosurgeons, the system demonstrated an intraoperative accuracy of 88.67% (41).

The efficacy of TSS is strongly influenced by tumor consistency. While soft PitNETs are generally amenable to complete resection, firmer tumors present greater technical challenges, frequently requiring adjunctive interventions such as extended surgical approaches or adjuvant radiotherapy (42). To address this variability, multiple deep learning models have been developed for preoperative tumor consistency prediction, including: 1) artificial neural networks (AUC = 0.710); 2) Extra Trees classifiers (AUC = 0.99); 3) Convolutional recurrent neural networks models (CRNNs, accuracy = 91.78%); 4) RF/SVM ensemble models (AUC = 0.90) (43–46). These predictive tools enable neurosurgeons to optimize surgical planning, thereby minimizing residual tumor burden and reducing postoperative complications.

#### 2.3.2 Gross-total resection

GTR represents a key surgical goal in TSS, with its feasibility dependent on several critical factors including cavernous sinus invasion, dural infiltration, tumor volume and consistency, growth pattern, and proximity to vital neurovascular structures. The Knosp classification system remains the clinical standard for evaluating tumor invasiveness and guiding surgical strategy (47). However, this system demonstrates limited sensitivity and specificity for microadenomas graded Knosp 1–3 (48). Recent advances in artificial intelligence have shown that deep neural networks significantly outperform conventional assessment methods in predicting GTR likelihood, achieving superior discriminative performance (AUC = 0.96) compared to both the Knosp system (AUC = 0.87) and logistic regression models (AUC = 0.86) (49). These results highlight the transformative potential of AI-based predictive models in optimizing preoperative planning and improving surgical outcomes.

#### 2.3.3 Postoperative remission

Deep learning models incorporating radiomic features show promising potential to assist neurosurgeons in preoperative treatment response prediction and personalized treatment planning for PitNETs (50). Recent studies have developed machine learning models to predict various remission outcomes in Cushing’s disease, including: 1) Early postoperative remission (SVM, AUC = 0.681; stacking model, AUC = 0.743); 2) Delayed remission (adaptive boosting [AdaBoost], AUC = 0.762); 3) Long-term cure (gradient boosting machine [GBM], AUC = 0.719) (51–53).

These results emphasize the prognostic value of several preoperative variables in predicting postoperative remission outcomes, including: (1) patient age, (2) presence of cavernous sinus invasion, (3) baseline ACTH levels, (4) tumor size and morphology, and (5) immunohistochemical ACTH staining characteristics.

Machine learning approaches have similarly been applied to predict surgical outcomes in acromegaly, demonstrating robust performance in forecasting both early remission (XGBoost, SHAP = 0.728; GBDT, AUC = 0.818) and delayed remission (XGBoost, AUC = 0.835; SHAP = 0.879) (54–56). Predictive modeling identified three key determinants of early remission: (1) preoperative GH levels, (2) patient age, and (3) tumor size. For long-term outcomes, the principal predictive factors were somatostatin receptor ligand (SRL) resistance status and preoperative tumor dimensions.

Deep learning approaches have also demonstrated utility in postoperative endocrine function assessment, enabling tailored follow-up protocols for patients with PitNETs. A recent comparative study evaluated six machine learning algorithms against conventional logistic regression for predicting hormonal outcomes in non-functioning pituitary macroadenomas. The AdaBoost model exhibited the strongest performance, with AUC values of 0.82 (postoperative hormonal decline), 0.74 (new-onset hormone deficiency), and 0.85 (hormone recovery) (57). Further advancing this field, an ensemble model integrating multiple predictive features—including (1) preoperative treatment history, (2) MRI characteristics (tumor volume, Knosp grade, and invasiveness), and (3) serum GH and insulin-like growth factor-1 (IGF-1) levels—achieved superior predictive accuracy for endocrine remission in acromegaly (AUC = 0.803) (58).

Visual impairment from chiasmatic compression represents a frequent complication of PitNETs, where surgical decompression remains the primary treatment modality. Postoperative visual recovery, however, demonstrates considerable interpatient variability, with key prognostic factors including: (1) patient age, (2) tumor dimensions, (3) symptom duration, (4) baseline visual function, and (5) retinal nerve fiber layer thickness (59, 60). Recent advances in predictive modeling have employed machine learning approaches to forecast visual outcomes. One study extracted radiomic features from preoperative optic chiasm MRI scans, comparing three algorithms for visual field recovery prediction. The SVM model achieved superior performance (AUC = 0.824) (61). A subsequent investigation developed seven distinct classifiers incorporating multimodal clinical and ophthalmologic parameters, with the integrated model demonstrating exceptional predictive capability (AUC = 0.911). SHAP analysis revealed three dominant predictors: (1) preoperative visual field integrity, (2) ganglion cell complex thickness, and (3) maximal tumor diameter (62).

#### 2.3.4 Recurrence

Despite optimal surgical management, PitNETs demonstrate a 10-20% recurrence rate, with several well-established risk factors: (1) aggressive histopathological subtypes, (2) persistent postoperative tumor remnants, (3) cavernous sinus invasion, and (4) extrasellar extension (63). Contemporary research has sought to improve recurrence risk prediction by incorporating these clinical parameters with advanced machine learning algorithms.

Initial investigations into recurrence prediction employed deep learning approaches analyzing radiomic features to forecast postoperative progression in non-functioning pituitary macroadenomas (NFPAs), demonstrating model performance with AUC values ranging from 0.78 to 0.96. Notably, the maximum intensity projection (MIP)-based model achieved exceptional predictive accuracy (AUC = 0.962) (64, 65). Subsequent research focused on Cushing’s disease recurrence prediction through comprehensive analysis of clinical datasets. Seven machine learning algorithms were evaluated using 17 clinically relevant variables, with the RF classifier demonstrating optimal performance (AUC = 0.781). Feature importance analysis revealed three predominant predictors: (1) patient age, (2) early postoperative serum cortisol levels, and (3) postoperative ACTH concentrations (66).

To enhance predictive performance, researchers developed a hybrid CNN-MLP architecture that synergistically combines clinical parameters with radiomic features. This multimodal approach achieved superior discrimination (AUC = 0.85), significantly outperforming unimodal models relying exclusively on clinical data (AUC = 0.73) or MRI-derived features (AUC = 0.83) (67). The relationship between tumor recurrence and histopathological characteristics has prompted development of more sophisticated predictive frameworks. A recent advancement incorporates both clinicopathological markers and radiomic signatures, enabling robust 5-year recurrence risk stratification for pituitary macroadenomas (AUC = 0.783). This integrated model demonstrates the value of combining histological classification with advanced imaging analytics for improved prognostication (68).

#### 2.3.5 Therapeutic response to medication

Prolactinomas represent the most common subtype of PitNETs, for which dopamine agonists (DAs) - including cabergoline and bromocriptine - constitute first-line therapy. In the majority of cases, DA treatment achieves multiple therapeutic goals: (1) tumor volume reduction, (2) PRL level normalization, (3) symptom alleviation, and (4) gonadal function restoration (35). However, 10-30% of patients demonstrate DA resistance (69), necessitating consideration of alternative interventions such as early surgical decompression or stereotactic radiosurgery (70). This clinical challenge underscores the importance of early identification of DA non-responders to facilitate timely treatment modification and improve clinical outcomes.

To address this clinical challenge, researchers developed a radiomics-based predictive model that combines conventional MRI features with machine learning algorithms. This approach achieved robust performance (AUC = 0.81) in identifying DA-resistant prolactinomas, enabling earlier therapeutic decision-making (71). A subsequent study implemented an advanced super-learner framework that integrated multiple deep learning classifiers. This model employed both the AUC and Matthews correlation coefficient (MCC) as complementary performance metrics, demonstrating exceptional predictive capability (AUC = 0.98; MCC = 0.93) for assessing DA treatment dependence. Feature importance analysis revealed temporal variations in predictive factors: baseline serum PRL levels were most influential for early treatment response, while 30-day post-treatment remission status served as the strongest predictor of long-term DA dependence (72).

Acromegaly demonstrates similar treatment challenges, with more than 95% of cases originating from GH-secreting PitNETs. While TSS constitutes first-line therapy, patients with persistent postoperative disease or those who are poor surgical candidates require pharmacological intervention. Somatostatin analogs (SAs) represent the cornerstone of medical management in these cases. The role of preoperative SA administration remains controversial. Although some evidence suggests potential benefits for surgical outcomes, SA resistance may lead to treatment delays and worse clinical prognosis (71). This clinical dilemma underscores the critical need for reliable biomarkers to predict SA responsiveness, which would significantly enhance personalized treatment strategies.

Recent advances in computational analysis have enabled more precise prediction of treatment responses in acromegaly. A machine learning framework incorporating quantitative texture analysis of T2-weighted MRI demonstrated strong predictive performance for SA therapy response in GH-secreting macroadenomas, achieving 85.1% classification accuracy with an AUC-ROC of 0.847 (73). Further refining predictive capabilities, an Extreme Gradient Boosting model was developed to anticipate SRL resistance. This model attained an AUC of 0.753, with feature importance analysis identifying three key predictive factors: (1) postoperative 3-month IGF-1 levels, (2) 3-month GH levels, and (3) histological classification as sparsely granulated somatotroph adenoma (55).

### 2.4 Complication

#### 2.4.1 Cerebrospinal fluid leakage

Cerebrospinal fluid (CSF) leakage represents a frequent complication following TSS, with an incidence of approximately 30% in patients with PitNETs. This complication carries significant clinical implications, potentially resulting in serious postoperative sequelae including persistent headaches, meningitis, and surgical site infections (74). The development of reliable preoperative predictive models for CSF leakage risk is therefore essential for optimizing surgical approach selection, enhancing perioperative patient management, and reducing associated healthcare expenditures.

Recent advances in machine learning have yielded several predictive models for postoperative CSF leakage in PitNET patients. Three distinct algorithmic approaches have demonstrated particular efficacy: (1) RF classifiers (AUC = 0.84), (2) Bayesian generalized linear models (GLMs) (AUC = 0.71), and (3) TensorFlow-based neural networks (AUC = 0.84) (75–78). These models consistently identified three key predictive variables: Hardy classification grade, previous transsphenoidal surgery, and patient age. A subsequent innovation employed advanced neuroimaging analytics, developing a two-dimensional convolutional neural network (2D-CNN) model based on extracted MRI features. This approach achieved superior predictive performance (AUC = 0.90), with class-activation mapping analysis localizing the CSF flow pathway as the most anatomically significant predictor of postoperative leakage risk (79).

#### 2.4.2 Delayed hyponatremia

Delayed hyponatremia represents a common postoperative complication following TSS, occurring in approximately 15% of cases (80). The condition predominantly develops due to the syndrome of inappropriate antidiuretic hormone secretion (SIADH) caused by surgical manipulation of the posterior pituitary gland. As one of the most frequent causes of unplanned 30-day readmissions, delayed hyponatremia substantially impacts both patient morbidity and healthcare resource utilization. The identification of robust predictive biomarkers is therefore essential for improving postoperative surveillance protocols, reducing length of hospitalization, and preventing potentially life-threatening complications (81).

Emerging machine learning approaches have enabled accurate prediction of delayed postoperative hyponatremia in patients undergoing TSS for PitNETs. Comparative analyses of various algorithms revealed that XGboost (AUC = 0.831) and RF (AUC = 0.798) models demonstrated optimal predictive performance (82, 83). Feature importance analysis identified four clinically significant predictors: (1) degree of pituitary stalk deviation, (2) preoperative measurable pituitary stalk length, (3) postoperative measurable pituitary stalk length, and (4) the magnitude of serum sodium concentration change between baseline and postoperative day 2.

#### 2.4.3 Diabetes insipidus

TSS for PitNETs carries a risk of posterior pituitary lobe injury, which may precipitate diabetes insipidus (DI) through disruption of arginine vasopressin (AVP) secretion. This complication manifests as a spectrum of water homeostasis disturbances ranging from transient polyuria to permanent dysregulation (84). The classic clinical triad of DI includes polyuria (>3 L/day), polydipsia, and hypernatremia. Without prompt intervention, these symptoms may progress to severe dehydration, neurological manifestations (lethargy, irritability), and significant deterioration in quality of life.

Recent advances in predictive analytics have enabled the development of machine learning models capable of anticipating postoperative DI in patients undergoing transsphenoidal surgery for PitNETs. Comparative evaluation of various algorithms revealed that RF models consistently demonstrate superior predictive capabilities, with one study reporting an AUC of 0.815 and identifying pituitary stalk invasion as the most significant prognostic factor (85). A subsequent multicenter study developed an integrated predictive framework incorporating both clinical and radiological parameters. The RF algorithm again outperformed other models, achieving exceptional accuracy (ACC = 0.882) and discriminative ability (AUC = 0.96). Feature importance analysis highlighted two key predictive clusters: morphometric changes in pituitary stalk dimensions and dynamic variations in anterior pituitary hormone profiles (86).

#### 2.4.4 Olfactory dysfunction

Olfactory dysfunction represents a frequent postoperative complication of TSS for PitNETs, with reported incidence rates varying from 10.5% to 44% across clinical series (87). This neurosensory impairment primarily stems from intraoperative trauma to olfactory-related anatomical structures, including the olfactory epithelium, nasal septal mucosa, and superior turbinates. The resultant anosmia or hyposmia can significantly impact patients’ quality of life, particularly affecting nutritional intake and environmental safety awareness.

A recent prospective cohort study developed and validated a machine learning-based predictive model for postoperative olfactory dysfunction following TSS. Among multiple algorithms evaluated, the RF classifier demonstrated optimal discriminative performance (AUC-ROC = 0.846, 95% CI: 0.812-0.879). This model enables preoperative risk stratification, early identification of high-risk patients, and implementation of targeted preventive strategies during the perioperative period. The robust predictive accuracy and clinical interpretability of this model support its potential integration into standardized postoperative care pathways (88).

## 3 Conclusions

AI has emerged as a transformative technology in the comprehensive management of PitNETs (**Figure 1**). It offers significant strengths including enhanced efficiency in tumor detection through deep learning-based imaging analysis, improved subtype classification as well as invasiveness prediction via multimodal data fusion (i.e., integrating radiomics with clinical parameters), and personalized surgical planning through AI-driven risk prediction models. These advancements hold promise for optimizing treatment strategies, mitigating complications, and refining prognostic assessment for PitNETs.

However, critical limitations and challenges must be explicitly acknowledged. The variability in population characteristics across different centers and the heterogeneity of imaging equipment (e.g., differences in MRI scanner manufacturers, protocols, and resolutions) necessitate extensive data harmonization and often significant model fine-tuning to ensure generalizability and robustness. Deployment in clinical settings faces substantial hurdles, including computational resource requirements, integration with existing hospital workflows and electronic health records (EHRs), regulatory approvals, and ensuring equitable model accessibility. Furthermore, unmet needs persist in modeling complex biological behaviors and specific clinical variables. Current AI models often struggle to adequately capture and predict aspects like subtle tumor invasion patterns not readily apparent on standard imaging, rare subtypes with limited data, nuanced hormonal dynamics, long-term treatment response variations, and the intricate interplay of molecular markers with imaging and clinical phenotypes (89, 90). Addressing these gaps is crucial for comprehensive predictive modeling.

Future research should therefore prioritize several key directions: (1) Developing more interpretable and robust algorithms capable of handling small-sample learning and inherent data variability; (2) Conducting large-scale, multi-center collaborative research that integrate detailed clinical characteristics, standardized imaging data, molecular profiles, intraoperative assessment and postoperative follow-up information; (3) Rigorously validating AI tools through prospective clinical trials to establish their clinical reliability and efficacy within the framework of evidence-based medicine; and (4) Actively addressing the practical challenges of clinical integration and accessibility. Collectively, overcoming these limitations and focusing on the unmet needs will be essential to expedite the transition of PitNET diagnosis and treatment towards truly precise, intelligent, and clinically impactful medicine.
