# Supplementary material for: Artificial intelligence-driven approaches in pituitary neuroendocrine tumors: integrating endocrine-metabolic profiling for enhanced diagnostics and therapeutics
Source: Front Endocrinol (Lausanne). 2025 Oct 16;16:1618412. doi: 10.3389/fendo.2025.1618412 (PMC12571619; doi:10.3389/fendo.2025.1618412)
Supplement: Supplementary file 1 [file Table1.docx]

**Supplementary Table 1:** Overview of AI-driven clinical applications in PitNET management.

| **Authors (Year)** | **Objective** | **Model Features** | **Sample Volume (Training/Testing)** | **Model/Algorithm** | **Best Performance** |
| --- | --- | --- | --- | --- | --- |
| Wang Y, Chen S, et al (2021) | To differentiate cystic PitNET from Rathke cleft cyst | MRI radiomics features | N=215 (172/43) | SVM, ANN, AdaBoost, RF | (ANN)  AUC=0.848  Sensitivity: 73.9%  Specificity: 80.0%  Accuracy: 76.7% |
| Bou-Nassif R, Reiner AS, et al (2024) | To differentiate between PitNET and normal gland | Immunohistochemical features | N=194 | CNN | Sensitivity: 96.1%  Specificity: 92.7% |
| Baysal B, Eser MB, et al (2022) | To distinguish prolactinoma from other PitNET subtypes | MRI radiomics features | N=130 | ANN | AUC=0.95  Sensitivity: 91%  Specificity: 98% |
| Zhang S, Song G, et al (2018) | To predict null cell adenoma among non-functioning adenomas | MRI radiomics features | N=112 (75/37) | SVM | AUC=0.8042 |
| Rui W, Qiao N, et al (2022) | To predict silent corticotroph adenoma among non-functioning adenomas | MRI radiomics features | N=302 (242/60) | LR, SVM, RF, GBM | (The ensemble algorithm)  AUC=0.927 |
| Feng T, Fang Y, et al (2022) | To accurately assess sellar floor invasion of PitNET | MRI radiomics features | N=1413 | CNN | AUC=0.98  Sensitivity: 96.4%  Specificity: 95.8%  Accuracy: 96.0% |
| Shu XJ, Chang H, et al (2022) | To make preoperative prediction of Ki67 labeling index status in PitNET | MRI radiomics features | N=261 (234/27) | KNN | Accuracy: 89.4% |
| Mao Z, Das A, et al (2024) | To localize critical anatomical structures in endoscopic pituitary surgery | Endoscopic images | Dataset: 635 frames obtained from 64 endoscopic pituitary surgery videos | CNN (“PitSurgRT” model) | Accuracy: 88.67% |
| Cuocolo R, Ugga L, et al (2020) | To make preoperative evaluation of pituitary macroadenoma consistency | MRI radiomics features | N=89 | RF (“Extra Trees Classifier”) | AUC=0.99  Sensitivity: 100%  Specificity: 87%  Accuracy: 93% |
| Staartjes VE, Serra C, et al (2018) | To predict gross-total resection after TSS for PitNET | MRI radiomics features | N=140 | DNN | AUC=0.96  Sensitivity: 94%  Specificity: 89%  Accuracy: 91% |
| Fan Y, Li Y, et al (2021) | To predict delayed remission in non-immediate remission patients with Cushing’s disease | MRI radiomics features and clinical features | N=201 (160/41) | LR, AdaBoost, GBDT, XGBoost, CatBoost | (AdaBoost)  AUC=0.762  Sensitivity: 70.0%  Specificity: 66.7%  Accuracy: 68.3% |
| Fan Y, Wang R, et al (2020) | To make prediction of TSS response for acromegaly | Clinical features | N=668 (534/134) | LR, RF, AdaBoost, GBDT, XGBoost | (GBDT)  AUC=0.856  Sensitivity: 84.7%  Specificity: 85.0%  Accuracy: 79.1% |
| Zhang Y, Luo Y, et al (2021) | To predict pituitary macroadenoma recurrence | MRI radiomics features and clinical features | N=168 (116/52) | ANN | AUC=0.783  Sensitivity: 82.6%  Specificity: 79.3%  Accuracy: 80.8% |
| Kocak B, Durmaz ES, et al (2019) | To predict response to somatostatin analogues in acromegaly patients with growth hormone-secreting pituitary macroadenoma | MRI radiomics features | N=47 | KNN | AUC=0.847  Accuracy: 85.1% |
| Mattogno PP, Caccavella VM, et al (2022) | To preoperatively identify patients at high risk for intraoperative CSF leakage | Clinical features | N=210 (165/45) | RF | AUC=0.83 |
| Behzadi F, Alhusseini M, et al (2024) | To preoperatively identify risk factors of intraoperative CSF leakage from preoperative MRI | MRI radiomics features | N=220 (154/66) | CNN | AUC=0.90  Sensitivity: 86%  Specificity: 93%  Accuracy: 92% |
| Lin K, Zhang J, et al (2025) | To predict the occurrence of delayed hyponatremia after TSS for PitNET | Clinical features | N=452 (361/91) | XGBoost, GBDT, RF, SVM, LR, KNN, NB | (XGBoost)  AUC=0.821 |
| Hou S, Li X, et al (2023) | To predict risk of diabetes insipidus after TSS for PitNET | Clinical features | N=232 (162/70) | LR, RF, SVM, GBDT | (RF)  AUC=0.815 |
| Chen, Yuyang et al (2024) | To predict arginine vasopressin deficiency following TSS for PitNET | MRI radiomics features and clinical features | N=452 (316/136) | LR, SVM, RF, GBDT, KNN, XGBoost | (RF)  AUC=0.96  Accuracy: 88.2% |
| Chen M, Li Y, et al (2024) | To predict risk of olfactory dysfunction after transnasal sellar pituitary tumor resection | Clinical features | N=158 (126/32) | BPNN, LR, GBDT, SVM, RF, XGBoost, AdaBoost | (RF)  AUC=0.846  Accuracy: 75.0% |

AI: artificial intelligence, AUC: area under curve, PitNET: pituitary neuroendocrine tumor, TSS: transsphenoidal surgery, MRI: magnetic resonance imaging, SVM: support vector machine, ANN: artificial neural network, RF: random forest, CNN: convolutional neural network, LR: logistic regression, GBM: gradient boosting machine, KNN: k-nearest neighbor, DNN: deep neural network, AdaBoost: adaptive boosting, XGBoost: extreme gradient boosting, CatBoost: categorical boosting, GBDT: gradient boosting decision tree, NB: naïve Bayes, BPNN: back propagation neural network.
